# Relative impact of key sources of systematic noise in Affymetrix and Illumina gene-expression microarray experiments

**DOI:** 10.1186/1471-2164-12-589

**Published:** 2011-12-01

**Authors:** Robert R Kitchen, Vicky S Sabine, Arthur A Simen, J Michael Dixon, John MS Bartlett, Andrew H Sims

**Affiliations:** 1 Applied Bioinformatics of Cancer Group, Breakthrough Breast Cancer Research Unit, Institute of Genetics and Molecular Medicine, Crewe Road South, Edinburgh, Edinburgh, EH4 2XR, UK; 2 School of Physics, University of Edinburgh, 10 Crichton Street, Edinburgh, EH8 9AB, UK; 3 Yale University School of Medicine, Department of Psychiatry, 300 George Street, Suite 901, New Haven, CT 06511, USA; 4 Endocrine Cancer Group, Edinburgh Cancer Research Centre, Institute of Genetics and Molecular Medicine, Crewe Road South, Edinburgh, EH4 2XR, UK; 5 Breast Cancer Research Group, Western General Hospital, Crewe Road South, Edinburgh, EH4 2XU, UK

## Abstract

**Background:**

Systematic processing noise, which includes batch effects, is very common in microarray experiments but is often ignored despite its potential to confound or compromise experimental results. Compromised results are most likely when re-analysing or integrating datasets from public repositories due to the different conditions under which each dataset is generated. To better understand the relative noise-contributions of various factors in experimental-design, we assessed several Illumina and Affymetrix datasets for technical variation between replicate hybridisations of Universal Human Reference (UHRR) and individual or pooled breast-tumour RNA.

**Results:**

A varying degree of systematic noise was observed in each of the datasets, however in all cases the relative amount of variation between standard control RNA replicates was found to be greatest at earlier points in the sample-preparation workflow. For example, 40.6% of the total variation in reported expressions were attributed to replicate extractions, compared to 13.9% due to amplification/labelling and 10.8% between replicate hybridisations. Deliberate probe-wise batch-correction methods were effective in reducing the magnitude of this variation, although the level of improvement was dependent on the sources of noise included in the model. Systematic noise introduced at the chip, run, and experiment levels of a combined Illumina dataset were found to be highly dependant upon the experimental design. Both UHRR and pools of RNA, which were derived from the samples of interest, modelled technical variation well although the pools were significantly better correlated (4% average improvement) and better emulated the effects of systematic noise, over all probes, than the UHRRs. The effect of this noise was not uniform over all probes, with low GC-content probes found to be more vulnerable to batch variation than probes with a higher GC-content.

**Conclusions:**

The magnitude of systematic processing noise in a microarray experiment is variable across probes and experiments, however it is generally the case that procedures earlier in the sample-preparation workflow are liable to introduce the most noise. Careful experimental design is important to protect against noise, detailed meta-data should always be provided, and diagnostic procedures should be routinely performed prior to downstream analyses for the detection of bias in microarray studies.

## Background

Increased adoption of high-throughput, whole-genome gene expression analysis technologies has led to an increased focus on the reliability of the experimental measurements they produce. Several recent articles have provided substantial evidence that systematic noise introduced as a result of batch-processing have a detrimental effect on data derived from microarrays [1-5]. Complete confounding of batches of array scans (even in the same laboratory, but at different times) with the variable under investigation can completely undermine reported results. For example, re-analysis of a study by Spielman *et al*. [6] revealed that samples from each of their studied populations were processed separately (between 1-3 years apart) and that after application of a standard batch-correction method [7] none of the genes initially reported remained significantly differentially expressed [8]. Several studies have attempted to assess reliability and consistency of array measurements and estimate potential sources of confounding systematic noise in an e ort to reduce these vulnerabilities [9,10]. The microarray quality control (MAQC) project was established to explore inter-platform and inter-laboratory consistency of microarray-derived gene expression datasets using two reference RNA samples [11], as well as consistency of differential expression estimates [12,13]. These studies reported generally good consensus between replicate samples across technologies and laboratories, however the latter found the greatest level of consistency in results-lists between platforms was achieved when probes were ranked by decreasing fold-change with a generous p-value cutoff [13]. Despite this finding, it is still the case that most published studies determine whether or not genes are differentially expressed based on the significance of statistical tests. Our own previous studies have reported compelling evidence for the existence of confounding batch effects in Affymetrix and Illumina experiments performed in the same laboratory using a single platform [14,15]. It has also been shown that batch effects can be mitigated using per-probe batch-correction methods such as mean-centring [14], distance-weighted discrimination (DWD) [16], or empirical-Bayes corrections [17].

Awareness of and protection from batch effects is of particular importance in meta- or re-analyses of existing data, collated from multiple sources, where the intention is to exploit increased statistical power to detect subtle differences in expression between well-defined phenotypes. Direct comparison and integration of gene expression data through re-analyses is a highly attractive option due to data repositories such as the NCBI Gene Expression Omnibus (GEO) [18] and Array Express hosted by the European Bioinformatics Institute [19]. Unfortunately appraising the quality of publicly available data is not a trivial task and, even when the submission guidelines are followed completely, it can be difficult to identify technical issues that may lead to systematic noise in subsequent analysis that potentially compromise results. It is important therefore to have an appreciation of the relative sources of systematic error throughout the experiment to prioritise which elements of meta-data should accompany published datasets and, subsequently, how to evaluate the experiment design to enable reliable re-analysis or integration of published data.

**Figure 1:**
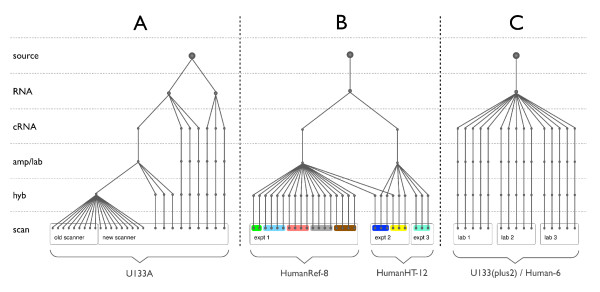
**Experiment designs**. Schematic comparison of the sample pre-processing used in the generation of **A**: the previously published MCF7 and MCF10A Affymetrix GeneChips, **B**: our Illumina Ref-8/HT-12 data, and **C**: the MAQC Illumina/Affymetrix data.

Given previously reported variation in various array technologies [14,15,20-23], we wished to quantify the relative noise introduced at various stages in the experimental process prior to array scanning; including the choice of experimental protocol, array-version, and study design. In addition, we assess different types of control sample in terms of their ability to model batch effects and also consider correlations to specific properties of array probes. In this study, we present an analysis of in-house Affymetrix (Figure 1A) and Illumina (Figure 1B) datasets in addition to those generated as part of the MAQC project [11] (Figure 1C) in order to assess the relative bias introduced in microarray results.

## Results

### Relative sources of systematic noise associated with various stages in the Affymetrix experiment workflow

**Figure 2:**
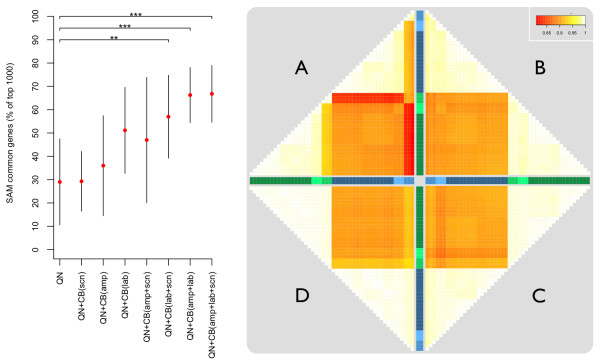
**Relative comparison of different batch effects in Affymetrix GeneChip data**. **LEFT**, Comparison of the numbers of differentially expressed genes in common between MCF7 and MCF10A triplicate samples depending upon what variables were included in the model used for the ComBat correction ('amp' = amplification, 'lab' = labelling, and 'scn' = scanner). Red points are mean counts, error bars are standard deviations, and significance of increased counts compared to no ComBat correction are indicated ('**' for p < 0:05 and '***' for p < 0:01, based on two-tailed t-tests). **RIGHT**, Pairwise Pearson correlation heatmaps of MCF7 and MCF10A samples compensating for 1, 2, or 3 sources of batch effect using ComBat. Green and blue colour-bars denote MCF7 and MCF10A samples, respectively. The lightest colours denote un-amplified samples, slightly darker are amplified samples, and the darkest colours are the scanner/labelling comparison. **A**, all data treated as a single group without batch-correction; **B**, batch-correction for amplification; **C**, batch-correction for amplification and an alternative labelling method; **D**, batch correction for amplification, labelling and different scanners used.

In order to establish the relative impact of different sources of systematic noise we compared the expression profiles and numbers of consistently differentially expressed genes when compensating for variation introduced at a single or at multiple steps in the Affymetrix GeneChip sample-processing workflow. The dataset, previously reported in [14], consisted of MCF7 and MCF10A samples for which technical replicates were available at several stages of sample preparation (Figure 1A). We previously observed that the variability introduced by different generations of array platform or amplification protocol was larger than that attributed to the choice of scanner used for image-capture. This was also reflected in the number of commonly differentially expressed genes across datasets (Eg. MCF7-amplified/MCF10A-unamplified vs. MCF7-unamplified/MCF10A-amplified), which were fewer between platforms/protocols than between scanners. In the current study, we found that when batch correction (using *ComBat *[17]) is performed to enable integration of these datasets, compensating for the bias introduced by amplification and labelling resulted in much greater consistency in gene-lists (46% and 66% average improvement) than correcting for the scanner (11% improvement), see Figure 2. Correction for significant sources of systematic error is required to improve the likelihood of consistently identifying differentially expressed genes from different datasets, but these approaches do not completely remove all sources of technical error.

**Figure 3:**
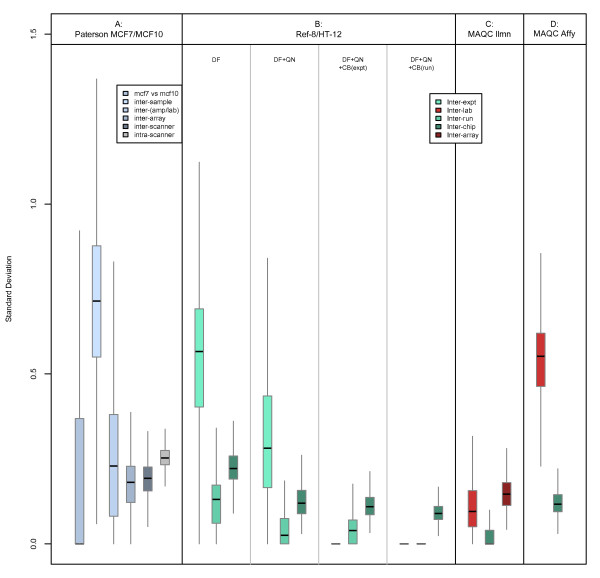
**Comparison of variance components in Affymetrix and Illumina data**. Comparison of MAQC and Paterson Affymetrix variance components. **A**: Probe-wise SD-estimates corresponding to several levels of technical variance (see figure key) in detection-filtered and quantile normalised MCF7/MCF10 expressions from the Paterson Affymetrix dataset. **B**: Probe-wise estimates of standard deviations (SD) corresponding to the inter-experiment (light blue), inter-run (dark blue), and inter-chip (green) technical variance in our UHRR Illumina data. The effect on these standard deviations following detection-filtering (DF), quantile-normalisation (QN), *ComBat *batch-correction by experiment (CB(expt)), and *ComBat *batch-correction by run (CB(run)) are shown. **C**: Probe-wise SD-estimates corresponding to inter-laboratory (pale blue), inter-chip (green), and inter-array (dark green) technical variances in detection-filtered and quantile normalised UHRR/UBRR expressions from the MAQC Illumina dataset. **D**: Probe-wise SD-estimates corresponding to inter-laboratory (pale blue) and inter-chip (green) technical variances in detection-filtered and quantile normalised UHRR/UBRR expressions from the MAQC Affymetrix dataset.

Variance analysis of this dataset provided a more detailed breakdown of the estimated biological and technical error introduced at the various stages of sample-preparation (Figure 3A). The majority of the probesets show far greater inter-sample variability than between the two cell-lines. Cell-line to cell-line (MCF7 vs. MCF10A) variability, mean-averaged over all probesets, contributed 9.6% to the total standard deviation; compared to 40.6% due to inter-sample variability, 13.9% due to amplification/labelling, 9.9% & 10.8% for inter array and inter-scanner, and 15.2% at the within-scanner/residual level. The arrays were preprocessed using the popular RMA algorithm for background correction followed by quantile normalisation (see methods), however not performing any background correction resulted in a similar relative variance pro le but noticeably reduced absolute variances (Additional File 1). Either with or without background correction, this error pro le is not at all dissimilar to error estimates obtained from the various stages of sample preparation prior to a qPCR experiment in which the sampling step is usually by far the most variable while the noise introduced during reverse-transcription is generally low, but can sometimes be larger than sampling error [24].

### Technical processing noise across Illumina Beadarray chip, runs, and experiments

Variance estimates were calculated for each of 15,757 probes at the inter-experiment, inter-run, and inter-chip level using a nested analysis of variance described in methods (Figure 1B, Figure 3B, and Additional File 2). The inter-experiment variance was the parameter with the greatest source of measurement noise, accounting for an average 61.7% of the total variation in reported gene expression. This is likely to be due to the use of a fresh round of amplification and labelling performed on the UHRR samples between experiments. Despite an overall reduction in the technical variation between probes, the fraction of the total variance contributed by each level (inter-chip, -run, and -experiment) was unaffected by standard, array-wise, quantile normalisation. Specific, probe-wise, batch correction greatly reduces the technical variation due to experiment and run (Figure 3B). The slight effect due to the within-batch variance moderation can be measured in the reduction in inter-chip SD after either run of *ComBat*. The high inter-chip variation, compared to inter-run variation, appears to be driven by the new samples run on the HT-12 chips as this was not observed in the standalone analyses of the Ref-8 data alone. As with the Affymetrix data we explored the effect of background correction on these variances (Additional File 1); the practice of correcting for background signal in Illumina arrays is not as common as when processing Affymetrix data so we compared variances between preprocessing using quantile normalisation with no background correction to those after using the *neqc *function in *limma *package, which uses the various negative control probes on an Illumina array to compensate for background signal prior to quantile normalisation. As with the Affymetrix arrays it is clear that performing additional background correction has little effect on the relative variance structures but generally inflates the magnitudes of the estimated variances.

A similar variance analysis was also performed, for comparison, using the MAQC Illumina Human-6 Expression BeadChip (v1) dataset [11]. Expression levels for 47,293 probes were subjected to the same detection-filtering criteria as our Ref-8 and HT-12 chips and the expressions of 21,896 surviving probes were quantile normalised prior to variance analyses. The design of the MAQC experiment only allowed for variance components to be estimated for the inter-laboratory, inter-chip, and inter-array levels. However, compared to the Ref-8/HT-12 inter-experiment standard deviations, the MAQC inter-laboratory standard deviations were much smaller; less than half as much on average (Figure 3C). This is likely to be a result of our use of different array-versions and widely different dates on which the arrays were processed (about 2 years). However it is noteworthy that the majority of the variance in the MAQC dataset was attributable to the intra-chip (inter-array) level and this is the only dataset, for which variance analyses were performed, in which this phenomenon holds. Variance analysis of the MAQC Affymetrix U133 Plus2.0 dataset (using 23,053 probes reported as 'present' across at least 80% of the samples) revealed a more familiar variance pattern, similar to that observed in our experiments, in which the majority of the variation is directly attributable to systematic differences between the different laboratories performing the experiments (Figure 3D). The estimated standard deviations at the inter-laboratory level in these data were similar to those observed at the inter-experiment level in the combined Ref-8/HT-12 data, as were the distributions of the coefficients of variation (standard deviation normalised by mean expression; data not shown).

**Figure 4:**
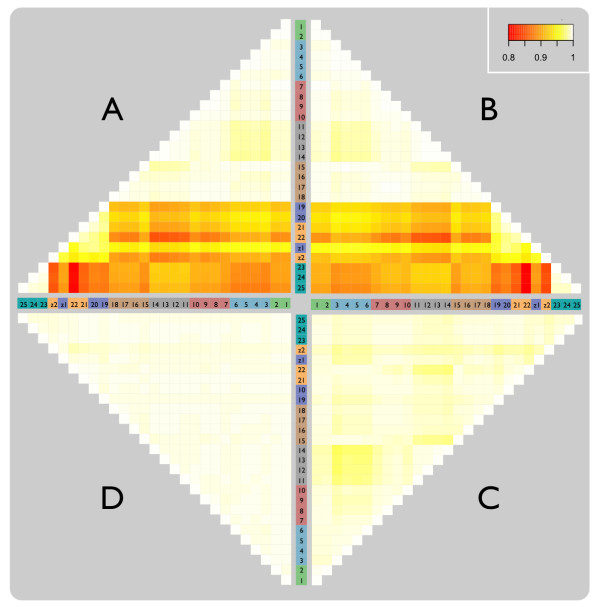
**Correlation heatmap of all replicate UHRR pairs**. Heatmap of Pearson correlations between replicate pairs of UHRR samples highlights the inter-experiment, inter-run, and inter-chip differences; particularly at the inter-experiment level. Red cells correspond to ~80% correlation and white to 100% correlation. Batches and sample numbers are consistent with the colouring and labelling in Figures 1 and Additional File 2. **A**: detection filtered (DF); **B**: DF & quantile normalised (QN); **C**: DF & QN &*ComBat*(by experiment); **D**: DF & QN &*ComBat*(by run).

The Pearson-correlation heatmaps in Figure 4 demonstrate that the majority of the variation between replicate UHRR samples in our Ref-8/HT-12 data was introduced as a result of the three separate experiments (see methods and Additional File 2). However, somewhat surprisingly, the poorest correlations exist between the two experiments involving the HT-12 chips (85.6% between runs 6/7 and run 8) as opposed to (90.4% between runs 1-5 and run 8). As previously observed, standard normalisation techniques based on array-wide intensity distributions, such as by quantiles or splines, do little to improve probe-wise correlations between replicate samples [15]. There is also an apparent band of poorer correlation corresponding to a single HT-12 chip in run 7 (chip 22). For consistency with the control samples hybridised to the Ref-8 chips, two replicates of the original UHRR were retrieved from storage and added to one chip in each run with the HT-12 chips in experiment 2. However, only a marginal improvement was observed in the correlation of these old UHRR samples compared to the freshly amplified and labelled replicates (C1-18 vs. Cz1-Cz2 = 92.8% compared to C1-18 vs. C19-22 = 91.2%). Although, again, the sample on chip 22 was more poorly correlated with the UHRR samples on the Ref-8 chips than that hybridised to chip 20, despite this chip appearing normal in all standard QC checks and internal controls except, perhaps, for slightly smaller bead-level standard errors compared to the other chips (see methods).

All UHRR data were subjected to batch-correction using *ComBat *and correction at two levels was assessed: experiment-wise and run-wise correction. Following *ComBat *correction by experiment, all pairwise correlations increased to approximately the same level observed in the quantile-normalised Ref-8 data obtained from the first experiment (Figure 4C). However, we found previously that despite high correlation between UHRR replicates the consistency between lists of statistically significant differentially expressed probes from duplicate sets of samples was poor without specific batch-correction [15]. Following *ComBat *correction by run, correlations in these new UHRR samples approach those observed in the original, *ComBat *batch-corrected UHRR samples from the first experiment (Figure 4D).

### Inter-batch calibrators: Comparing UHRR with pools of tumour sample RNA

**Figure 5:**
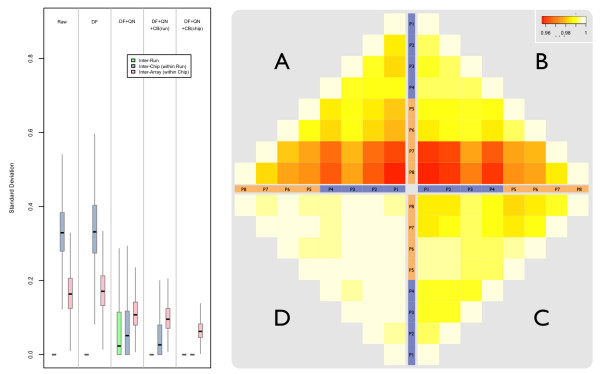
**Correlation heatmap of all replicate pool pairs**. **RIGHT**: Heatmap of Pearson correlations between replicate pairs of pooled-tumour control samples highlights the inter-run and inter-chip variation; particularly at the inter-chip level. Red cells correspond to ~96% correlation and white to 100% correlation. Batches and sample numbers are consistent with the colouring and labelling in Figures 1 and Additional File 2. **A**: detection filtered (DF); **B**: DF & quantile normalised (QN); **C**: DF & QN &*ComBat*(by run); **D**: DF & QN &*ComBat*(by chip). **LEFT**: Variance estimates at various levels in replicate pooled-controls hybridised to the HT-12 chips used in experiment 2. Highlights the effect of various normalisation procedures on these variance estimates; as before, such procedures include detection-filtered (DF), quantile normalised (QN), *ComBat *corrected by run (CB(run)), and *ComBat *corrected by BeadChip (CB(chip)).

Two controls of pooled clinical breast-tumour RNA were run on each of the four BeadChips in experiment 2 (samples P1 through P8 in Additional File 2). One pool was created from a mix of all seven pre-treatment samples run in this experiment and the second pool was created using all seven post-treatment samples (see methods). Results of pairwise Pearson-correlations between these pooled samples identified a large difference between chip 22 and the other three chips used in this experiment (Figures 5A &5B), consistent with that seen with the UHRR. No obvious differences were observed in the correlations between the different pools composed of either pre- or post-treatment RNA. In general, the correlations appeared similar to those observed between replicate UHRR samples illustrated in Figure 4. As noted before, quantile normalisation does little to remedy the poor correlation between chip 22 and the other chips, but this is remedied with *ComBat *by treating the batches either as runs (Figure 5C) or, slightly better, as separate chips (Figure 5D).

Compared to the UHRR samples in experiment 1 (Figure 3B), variance components estimated by nested-anova between the pooled tumour samples (Figure 5) in experiment 2 both show a large inter-chip/intra-batch effect. The larger magnitude of variation supports the increased inter-chip component in UHRR and it is clear from both the correlations and the variance estimates of both the UHRR and tumour pools that the variance in the HT-12 data is driven by a particular chip (chip 22), rather than between runs as was the case in the Ref-8 arrays.

Gene expression in this small group of samples was rather varied, resulting in only 5 probes significantly differentially expressed following treatment that also survived multiple-testing correction at q < 0.05; the same 5 probes appeared in every results list regardless of normalisation or batch-correction. Despite the apparent low level of biological variation between the pre- and post-treatment samples and small number of significantly differentially expressed genes, both the UHRR and the pools accurately reproduced the fold-changes between duplicate tumour samples across the two runs (Additional File 3). We calculated the differences in expression across the two batches for all duplicate pairs of tumour samples and also between the UHRR/pool replicates. The correlation between differences observed in the tumour duplicates and either the UHRR or pool controls increased with the magnitude of the difference, although both the pre-and post-tumour pools were found to be significantly better correlated compared to UHRR (on average by 3.9% and 4.3%, using one-sample, two-tail t-tests p < 5.9 * 10^-9 ^and p < 3.4 * 10^-9^, respectively). We also performed an exhaustive comparison of the fold-change in expression due to the different runs between the replicate pools of pre-treatment tumour RNA and each of the fold-changes between duplicate tumour samples (Additional File 4). In many cases there was strong agreement between absolute expression differences observed in the pool and the individual tumour duplicates, however such agreement is not necessary in order for the effect to be removed by *ComBat*, or alternative correction methods, as the absolute magnitude of the effect is explicitly normalised during the correction procedure.

### Properties of the probes with respect to batch variation

In order to assess if batch effects are uniform across all genes, we investigated whether the properties of the probes themselves were affected by different sources of systematic error (see methods). Several descriptive statistics were selected to serve as measures of probe- and mapping-specific properties that could conceivably influence probe expression [25-28]. These included compositional properties such as the guanine and cytosine (GC) nucleotide content as well as mapping properties such as the position of the probe as a fraction of the total length of the target gene, the number of (known) transcripts consecutively probed, and the average number of exons within the probed gene; with the number of known transcripts and number of exons acting as proxies for gene complexity and size, respectively.

**Figure 6:**
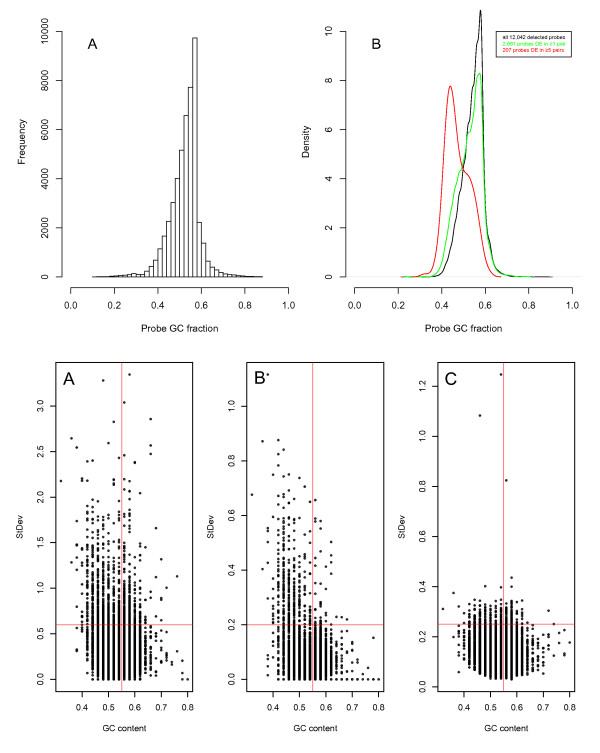
**Batch effect and probe GC content**. **TOP**: **A**, histogram of GC content over our Illumina HT-12 probes; **B**, gaussian-smoothed probability density distribution (black, N = 48,803). Union of probes more than 2-fold up- or down-regulated due to the batch effect in any of the 14 duplicate tumour-sample pairs from Additional File 4 (green line, N = 2,661). Union of probes more than 2-fold up- or down-regulated due to the batch effect in more than 5 of the duplicate tumour pairs (red line, N = 207). **BOTTOM**: Plots of probe CG-fraction against standard deviation estimated at the inter-experiment (**A**), inter-run (**B**), and inter-chip (**C**) levels in our combined Illumina Ref-8/HT-12 dataset. Red lines denote the cutoff s used in chi-squared analysis at each level.

The distribution of GC-content over the Illumina HT-12 probes (Figure 6A-top) clearly shows that they have been designed with a bias such that the nucleotide distribution of the probes themselves favour a greater-than-random fraction of GC-nucleotides. The GC-fraction distribution of the 12,042 probes used in the analysis of run-induced fold change is plotted as a Gaussian-smoothed probability density in Figure 6B-top. Also plotted is the distribution of the subset of these probes found to be more than 2-fold up- or down-regulated due to the batch effect in any of the 14 duplicate sample pairs. These two distributions are very similar, suggesting that a large number of these technical effects are random fluctuations independent of GC-content. However the distribution of any probe found to be more than 2-fold up- or down-regulated due to the batch effect in at least 6 of the duplicate sample pairs was significantly biased towards a lower GC-fraction (Figure 6B-top; χ^2 ^p-value < 2.2 * 10^-16^). Taking this further, we correlated probe GC-fraction with error estimates from the Ref-8/HT-12 UHRR replicates (Figure 3B), revealing highly significant enrichment for higher standard deviations (SD) at lower GC-fractions (< 0.55) at the inter-experiment (Figure 6A-bottom; χ^2 ^pVal < 5.3 * 10^-8^) and inter-run (Figure 6B-bottom; χ^2 ^pVal < 2.2 * 10^-16^) levels, but not at the inter-chip level (Figure 6C-bottom). No significant association was observed between low-signal probes and GC-content, however a small but significant enrichment for highly-signal-low-GC probes was found (Additional File 5).

We also compared probe GC content with our variance estimates from the MAQC Illumina and Affymetrix datasets, both of which have a similar overall distribution of probe-GC content, skewed in favour of higher GC fraction (data not shown). A significant enrichment for low-GC-high-SD probes was again observed, both at the inter-laboratory level (χ^2 ^pVal < 9.8 * 10^-8^) and at the inter-chip level (χ^2 ^pVal < 2.1 * 10^-4^). As with the Ref-8/HT-12 data, there was no significant enrichment at the lowest level between replicate arrays. To determine whether this low-GC-high-SD association was resolvable between biological as well as technical replicates, we chose a subset of pre-and post-treatment samples from our Illumina Ref-8 dataset (these were pre-treatment biological replicates from run 3 and non-duplicated post-treatment biological replicates from run 5 to avoid confounding due to inter-run variation). Despite a slightly greater number of probes with low-GC-high-SD in both the pre- and post-treatment sample-sets, the trend was not significant (data not shown).

The subset of pre-treatment biological replicates from run 3 and non-duplicated post-treatment biological replicates from run 5 were also used to test for any association between probe-mapping properties and batch effects. For each probe, the standard deviation between the pre-treatment samples and, separately, the post-treatment samples were assessed against probe position in the target gene, number of concurrently probed transcripts, and the number of exons in the target gene. The distribution of probe-location as a fraction of gene length among the Ref-8 probes is shown in Additional File 6. After adjusting for the strand of the target, there was no significant association for increased standard deviations of probes proximal to either the 3' or 5' end. To test whether probe-mapping may correlate with variation between replicate cRNA-syntheses, a similar analysis was performed using the MAQC Illumina dataset. These probes have a distribution similar to the Ref-8 arrays and the analyses again revealed no significant enrichment for estimated standard deviation due to probe position (Additional File 7). It is therefore likely that such probe-mapping effects can only be resolved when probes target different regions of the transcript, rather than for probes targeting the same region being affected by biological or technical variability.

## Discussion

Microarrays represent a powerful means of rapidly assessing genome-wide expression patterns. Unfortunately confounding technical variation and systematic error in array technologies presents a major obstacle to their adoption for clinical diagnostics in humans. These factors are rarely documented, poorly understood, and their implications for experimental and clinical utility of microarrays frequently ignored [1,4]. Building on previous investigations of technical variation between replicate RNA samples from breast tumour biopsies, this extended study used both Illumina and Affymetrix arrays to explore the reliability of reported expressions across a variety of experiment designs. Re-analysis of our MCF7 and MCF10A Affymetrix datasets demonstrated that not compensating for one batch effect, such as the use of a different scanner (Eg. if the information was not available) would have a much smaller effect on the numbers of genes identified as commonly differentially expressed, than another batch effect (such as the labelling method or if RNA was amplified).

Using a large combined dataset of conserved, reliably-detected probes on Illumina Ref-8 and HT-12 BeadChips (experiments 1 through 3) we found that the correlation between replicate UHRR hybridisations were consistently poorer than correlations previously reported using the Ref-8 data alone [15]. Interestingly, we found that labelled UHRR samples from the original experiment, which were stored at -80°C for approximately two years, hybridised to two arrays on the new HT-12 chips correlated better with the original Ref-8 samples than did freshly prepared UHRR replicates. This suggests that even long periods of frozen storage and additional freeze-thaw cycles introduce less noise into experimental measurements than that inherent in creating a new preparation of labeled cRNA, even from the same RNA source.

As in our previous investigation [15], quantile normalisation did little to improve correlation between the UHRR replicates across the Ref-8/HT-12 dataset. However specific batch-correction using *ComBat *[17] once again significantly improved correlations and is a valuable tool for removing systematic error introduced between experiments and/or processing runs. The inter-chip variation in the new HT-12 datasets was almost double what it was in the Ref-8 dataset and due to this increase in inter-chip variation and high-levels of inter-experiment variation, the inter-run variation in the combined dataset was largely obscured. However, as we have previously seen, inter-experiment and inter-run variances were largely eliminated following *ComBat *corrections.

Variance estimates using the MAQC Illumina dataset were smaller in magnitude to the variances obtained from our Ref-8 data, revealing a surprisingly high level of reliability between the three laboratories that performed these experiments. In contrast, the MAQC Affymetrix dataset was found to be far more variable than their Illumina data but similar to the magnitudes of variance observed in both our combined Ref-8/HT-12 dataset and our Affymetrix dataset. The reason for the low variation in the MAQC Illumina data is unknown, especially since their study design deliberately split the sample replicates before cRNA synthesis; a much earlier stage in the sample-prep workflow than our replicates (which were split after amplification and labelling). It is possible that the small number of laboratories (three, in total) performing the MAQC Illumina hybridisations produced highly concordant data completely by chance, while the larger number of laboratories (six, in total) performing the Affymetrix experiments provided a more realistic reflection of the technical variation in these data.

### Pooled sample vs. UHRR as batch-effect calibrators

Several studies have found the use of replicate control samples such as UHRR to be a useful standard in microarray experiments, suitable for monitoring expression consistency within and across a variety of genome-wide expression platforms [29-32]. However, such commercial controls are deliberately generic and deficiencies have been reported in terms of how well they represent specific cell types [33]. Clearly UHRR is not representative of breast tumour RNA and therefore carries no guarantee of expressing RNAs that may be variably expressed in the specific subset of genes changed in breast tumour tissue. Therefore, in terms of compensating for confounding technical variation, the very probes for which the correction is most important are those that are most neglected in the UHRR controls.

Unfortunately the relatively small degree of legitimate biological differential expression between the pre-and post-treatment tumour biopsies provided little opportunity to assess the relative performance of UHRR and pooled batch calibrators on the consistency of reported differentially expressed probes. However, compared to the UHRR, the pooled tumour RNA controls were shown to more faithfully emulate the individual shift in expression between tumour technical-duplicates as a result of variation introduced between runs and between chips. Had it been possible to identify more legitimately differentially expressed probes between pre- and post-treatment samples, the pooled RNA would almost certainly have made for a better batch-calibrator during *ComBat *correction than the UHRR controls. If a similar pooled calibrator was used in our previous study it seems reasonable to speculate that the consistency between the gene-lists reported as significantly differentially expressed would have been noticeably higher than the 74.1% achieved using UHRR calibrators.

### Batch effects relative to probe position and composition

We took the opportunity to assess compositional properties of the probes as a potential explanation/surrogate for the technical effects observed in the Ref-8/HT-12 data. A highly significant trend in favour of low-GC content was identified in the core set of probes consistently affected by inter-run and inter-chip variation between sample duplicates. A similar, but less significant, enrichment for low-GC-high-SD probes was also observed in the MAQC Illumina dataset. This suggests that the magnitude of error introduced due to low probe GC-content is sufficiently great that it is resolvable between the replicate cRNA preparations assessed in the MAQC study. A similar observation regarding probe GC-content and expression consistency was recently reported in a comparison of RNA preservation protocols, using matched samples, in terms of the effect on results of downstream expression analyses [34]. A further correlation of probe composition, specifically with respect to GC-content, has been reported previously in a spike-in experiment using Illumina BeadChips [35], in which it was found that probes with high-GC content tended to have a higher than expected signal intensity, but probes with lower than average GC content had inflated differential expression statistics. We found no such association between low-expression and GC-content, however we did observe a low-signal-low-GC association; therefore the notion of inflated differential expression stats for low-GC probes is supported not by probe intensity, but through greater variability in our data. The low-GC effect is likely to be related to thermodynamic properties of hybridisation favouring high-GC probes/targets, a supposition that is rational given the deliberate high-GC bias in the design of the Illumina probesets. Probes with low GC-content appear to be inherently more vulnerable to systematic error but, although highly statistically significant, the magnitude of this variation in our data was small relative to that between biological replicates. It is therefore somewhat unlikely that such variation would pose a threat to the accurate classification of samples and, even in an experiment in which groups of biological replicates are poorly distributed across chips and runs, is also unlikely to be a serious confound to statistical tests for differential expression.

The proximity, with respect to the target transcript, of probes has been reported to strongly influence the correlation of expression measurements between technologies [36]. The analyses performed here were designed to assess whether such probe-transcript mapping influenced expressions reported by the same platform, however no such correlation was observed either between biological or technical replicate samples. A more thorough analysis of the MAQC datasets would provide further insight into any relationship between probe-location and expression between a variety of different platforms and sample-preparation procedures.

## Conclusions

The key source of systematic error in any given microarray experiment is unpredictable, but can generally be attributed to RNA extraction and, to some extent, labelling and amplification protocols. The results presented here, including those derived from external data sources, suggest that it is not recommended to analyse individual test samples (e.g. to then try and classify), but instead to run several at the same time to get a better estimate of the experimental variation in order to be better equipped to compensate for it.

Sound experimental design is of critical importance to avoid confounding systematic variation. Randomisation of samples over arrays on each BeadChip and careful blocking of samples from each group of interest within and across runs is necessary to protect against confounding systematic error. In situations where this is not possible, detailed meta-data should be preserved for each array that includes, at the very least, the date and time of each hybridisation and scan. In either case, diagnostic procedures such as PCA or SVA should be routinely performed prior to downstream array analyses.

## Methods

### Samples and Arrays

RNA was extracted using the RNeasy Mini Kit, including RNase-Free DNase treatment (Qiagen, Crawley, United Kingdom) from paired breast tumour tissue, alternatively a Universal Human Reference RNA (UHRR; Stratagene, Stockport, United Kingdom) was used. RNA was amplified and biotinylated using Illumina TotalPrep RNA Amplification Kit (Ambion) and quantified on an Agilent 2100 Bioanalyser. 750 ng cRNA per sample was hybridised to Illumina Human Ref-8 v2 or Human HT-12 v3 expression BeadChips (Illumina, Cambridge, United Kingdom) using the Whole-Genome Expression Direct Hybridisation kit (Illumina) and scanned with a BeadStation 500GX (Illumina). Duplication of the clinical samples was performed after labelling and labelled samples were stored as per the manufacturer's recommendations.

In addition to previously described samples [15,37], four Illumina HT-12 (v3) BeadChips were used in a follow-up experiment, described as 'Experiment 2' (Figures 1B and in Additional File 2). These chips were processed in pairs over the course of two days (referred to as runs). A single UHRR replicate, from a fresh preparation, was hybridised to each chip ('C19'-'C22'). Two replicates of the original UHRR from the original study were retrieved from storage at -80°C and one added to a chip on each run ('Cz1' and 'Cz2'). Pools of pre-treatment and post-treatment tumour RNA were created from the clinical samples and each pool was then hybridised to each chip. Three further Illumina Human HT-12 (v3) BeadChips were processed over the course of a single day, described as 'experiment 3' (see Additional File 2). Additional UHRR samples were included in two groups of replicates corresponding to different labelling protocols; the first group of samples was obtained from the same amplification and labelling (Ambion) as the UHRR samples used in experiment 2 ('C23'-'C25'). The second group of samples was labelled using the NuGen amplification kit (NuGen) ('Cn1'-'Cn4'; Figure Additional File 2). The remaining samples on these BeadChips were not analysed within this study.

Methods for generating the MCF7 and MCF10A triplicate Affymetrix U133A data can be found in the original publication [14]. Methods for the MAQC Illumina Human-6 Expression BeadChip (v1) and Affymetrix U133 Plus2.0 array hybridisations are provided in [11]. All raw gene expression les and clinical annotation generated in this study are publicly available from the caBIG supported Edinburgh Clinical Research Facility Data Repository https://catissuesuite.ecmc.ed.ac.uk/caarray/ and on request to the corresponding author.

### Statistical Methods

Quality control analyses revealed no systematic or individual problems with the Affymetrix or Illumina data at the array level as assessed by the array-level distributions of signal intensities (Additional File 8). For the Illumina Ref-8 and HT-12 arrays we also assessed the noise distributions between replicate beads as large differences in measurement precision might cause problems with the interpretation of results derived from the combined datasets; however both the bead standard errors and bead-representation on both Illumina chip-types were very similar (Additional File 8).

#### Handling of Illumina data

All Illumina array scans were initially processed using Illumina's *BeadStudio *software using default parameters, including removal of local background signal in the captured images but without background normalisation.

To perform reliable correlation and variance analyses simultaneously on both Illumina chip-types, we identified and retained only the 15,757 probes with exactly conserved sequences between Ref-8 and HT-12 that also mapped uniquely to the genome. Probes were re-mapped to the human genome (NCBI build 37) using *Bowtie *[38] (v.0.12.7) allowing for no mismatched bases in the alignment. Alignment annotation, for example the position of the probe within the host gene, was provided by in-house software and the NCBI RefSeq annotation database [39]. The 309 probes common to both the Ref-8 and HT-12 chips that aligned to the genome but fell within intergenic regions (or those that, for whatever reason, were not covered by the RefSeq annotation) were considered good and retained, along with the 15,448 annotated intragenic probes, for further analyses. All correlations, unless otherwise stated, are based on Pearson's product-moment and are reported as percentages where 100% is a perfect correlation.

These 15,757 uniquely mappable probes, represented on both Ref-8 and HT-12 chips, were further filtered (unless stated otherwise) using the detection confidence reported by *BeadStudio*- determined for each probe based on the expression of the internal control probes, local background intensity, and the uniformity of the reported intensity of the bead. The filtering was performed prior to quantile normalisation such that probes with a detection confidence less than or equal to 95% in more than 20% of the samples were removed from further analysis.

All Illumina data were detection-filtered and normalised together (8,948 of the conserved probes passed the detection-filter) before assessment by pairwise Pearson correlation. Results of pairwise Spearman rank correlations following separate filtering and normalisation of the Ref-8/HT-12 chips were very similar (data not shown). The globally filtered/normalised data were used in all subsequent UHRR analyses for consistency with our previous results and for a more reliable interpretation of nested variance analyses.

#### Handling of Affymetrix data

Affymetrix data were processed in *R *using functions contained within the *affy *package. Expression values were produced from probe in intensities using the *expresso *function with, unless stated otherwise, *RMA *background correction, quantile normalisation, and median-polish summarisation over probesets. Following this preprocessing, probes were filtered such that those reported 'absent' in more than 20% of the samples were removed.

#### Variance estimation

We applied a linear additive model to the Affymetrix and Illumina expression data on the log-scale to estimate the specified variance contributions. These contributions are assumed to be independent and randomly drawn from log-normal distributions. As all factors meet in unique combinations a nested, or hierarchical, variance model is individually applied for each gene. Models of this kind are formally defined in [40] and have previously been used in the context of gene-expression experiment design [24,41]. Variance estimates in all analyses described herein were performed using the REML procedure implemented in the *nlme *package in R [42-44]. In all mixed models the biological variables such as different cell-lines in the Paterson dataset and the UHRR/UBRR dilutions in the MAQC dataset were treated as fixed effects and all downstream sample-processing levels treated as random effects. The percentages reported in reference to the variance structures, for example in Figure 3, were calculated based on the mean standard deviation, of all linear models over all probes, attributed to each level.

#### Batch correction

The main difference between standard array normalisation and batch-correction is that the latter does not make the assumption that all probes are affected equally by batch-effects and, as such, performs individual adjustments on each probe across all samples. The ComBat approach, as discussed in detail in [17], is a somewhat involved process, but can essentially be summarised in three stages. First, using a method similar to autoscaling, the data over all genes are standardised so that they all have similar mean and variance. This step compensates for the different expressions and variations of the various probes/genes that would otherwise bias the batch effect estimates. Next the mean and variances of all samples in each batch, over all probes/genes, are estimated using a linear model and these parameters constitute the first prior-distribution. Next, independently for each gene, the sample mean and variance is estimated for each batch and are used to estimate the parameters of the additive and multiplicative noise distributions using the method of moments; these constitute the second prior-distributions. Finally, using the parameter estimates for these two prior distributions along with Bayes theorem, posterior distributions for each of the additive and multiplicative noise distributions are calculated; final values of these batch effect parameters are estimated as the expected values of the posterior distributions. This empirical Bayes procedure allows information from all genes to be used to estimate batch effects for each gene, providing more stable estimates than the standard sample mean and sample variance.

#### Differential expression

Gene expression changes were compared before and after treatments and between responders and non-responders using Bioconductor [45] algorithms implemented in the statistical programming language, *R *(v.2.12.1) [46]. Illumina probe pro le expression data were normalised by quantiles and corrected for batch processing effects using *ComBat *[17]. Genes differentially expressed between paired pre- and post-treatment samples were identified using *limma *(v.3.6.9) [47] and *SAM *(v.1.28) [48]. For the analysis using *limma*, empirical Bayes variance shrinkage was employed and genes were defined as being differentially expressed after satisfying a minimum fold-change of ± 1.5 and a maximum, Benjamini-Hochberg adjusted, p-value of 0.01. For the *SAM *analysis, the differentially expressed genes were selected, following 100 permutations, at a maximum predicted false discovery rate of 5% and the same minimum fold-change of ± 1.5.

R scripts and data archives can be downloaded from. https://catissuesuite.ecmc.ed.ac.uk/caarray/

## Authors' contributions

RRK and AHS conceived the study, performed the analyses, and drafted the manuscript; JMD supervised the tumour biopsies; VSS performed the sample-prep prior to the array analyses; and AAS, JMSB, and AHS supervised and funded the work. All authors read and approved the final manuscript.

## Supplementary Material

Additional file 1 **Supplementary material S1**. Comparison of estimated variance components in Affymetrix and Illumina data with and without background correction as part of the array pre-processing. Click here for file

Additional file 2 **Supplementary material S2**. Illustration of our Illumina Ref-8 (experiment 1) and HT-12 (experiments 2 & 3) BeadChips, processed in eight batches (also referred to as 'runs') corresponding to the different days on which the samples were hybridised and scanned. UHRR samples are labelled as C1-25. Replicate breast tumour clinical samples are identified with a suffix of 'a' through 'd'. The pre- and post-treatment biopsy samples are identified by a triangle to the left and right of the sample IDs, respectively. Click here for file

Additional file 3 **Supplementary material S3**. Correlation of expression change as a result of inter-run and inter-chip technical variation between UHRR and pooled controls with tumour duplicates. Tumour duplicates (individually plotted) are arranged on the x-axis to be close to others processed on the same BeadChip. UHRR and both types of pooled-control (comprised of pre- and post-treatment tumour RNA, respectively) are correlated more strongly with individual tumour duplicates in which one 'half' of the duplicate was processed on the outlying chip 22. Pooled-controls also consistently score slightly higher correlation than UHRR. Click here for file

Additional file 4 **Supplementary material S4**. Scatter plots of fold-changes between tumour duplicates and replicate pools. These plots each show the magnitude of the change in expression between technical replicates introduced by the runs and show how well correlated such changes are between the Pool/tumour-duplicates. In the figure, the pre-treatment tumour duplicate are coloured blue and post-treatment are green; probes that are differentially regulated, up or down, at least two-fold due to the different runs are highlighted in each plot (red points). Note that most of the samples with a duplicate on chip 22 are subject to a greater magnitude of variation than samples with a duplicate on chip 21. Click here for file

Additional file 5 **Supplementary material S5**. Plot of GC-content vs. (1/probe signal). Click here for file

Additional file 6 **Supplementary material S6**. Distribution of MAQC Illumina probes as a fraction of target gene length. Light blue points are used for probes that mapped to the antisense DNA strand and dark blue for the sense strand. Left plot represents the fraction of target gene length in terms of 3' and 5' coordinates on each strand while the right plot is 'normalised' for the anti-sense strand and plots the fraction in terms of absolute position along the DNA molecule. Click here for file

Additional file 7 **Supplementary material S7**. Probe position against probe standard deviation: Plots of probe position against probe standard deviation estimated at the inter-laboratory (**A**), inter-chip (**B**), and inter-array (**C**) levels in the MAQC Illumina dataset. Light and dark blue points again identify probes that mapped to the antisense and sense strands, respectively. Click here for file

Additional file 8 **Supplementary material S8**. Quality control metrics over all of our Affymetrix and Illumina arrays including array-level intensity distributions, Illumina bead-standard errors, and bead-representation distributions between the two Illumina array-versions. Click here for file
