# Supplementary material for: Relative impact of key sources of systematic noise in Affymetrix and Illumina gene-expression microarray experiments
Source: BMC Genomics. 2011 Dec 1;12:589. doi: 10.1186/1471-2164-12-589 (PMC3269440; doi:10.1186/1471-2164-12-589)
Supplement: Additional file 1 — Supplementary material S1. Comparison of estimated variance components in Affymetrix and Illumina data with and without background correction as part of the array pre-processing. [file 1471-2164-12-589-S1.PDF]

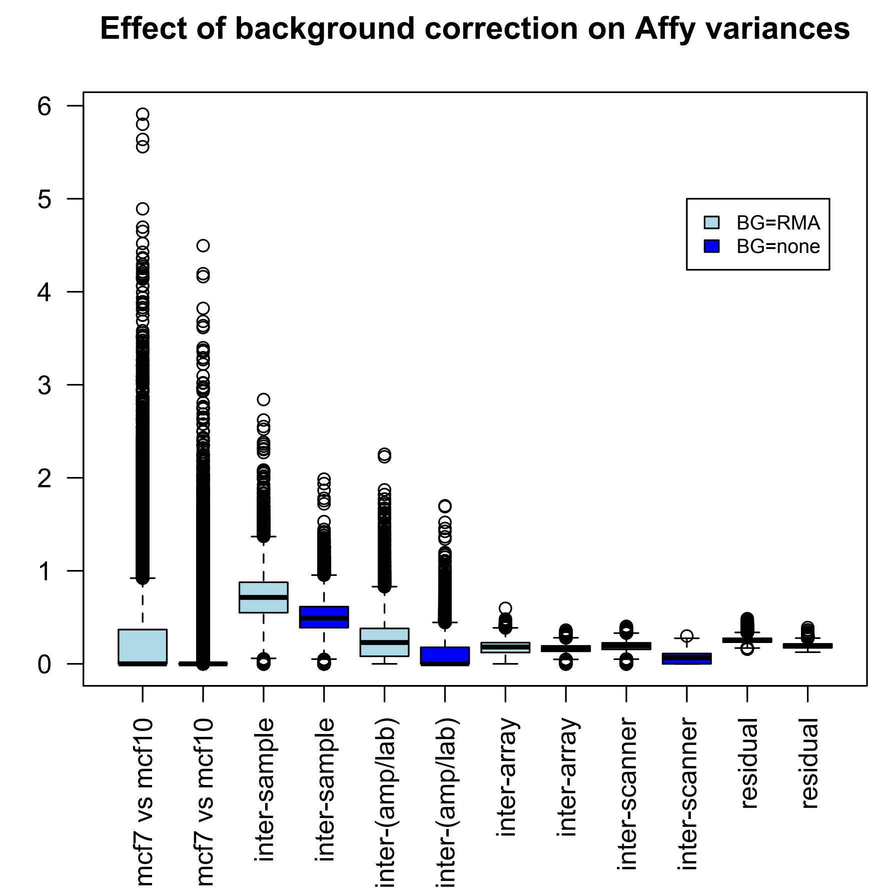
Effect of background correction on Affy variances
BG=RMA
BG=none
0
1
2
3
4
5
6
mcf7 vs mcf10
mcf7 vs mcf10
inter-sample
inter-sample
inter-(amp/lab)
inter-(amp/lab)
inter-array
inter-array
inter-scanner
inter-scanner
residual
residual

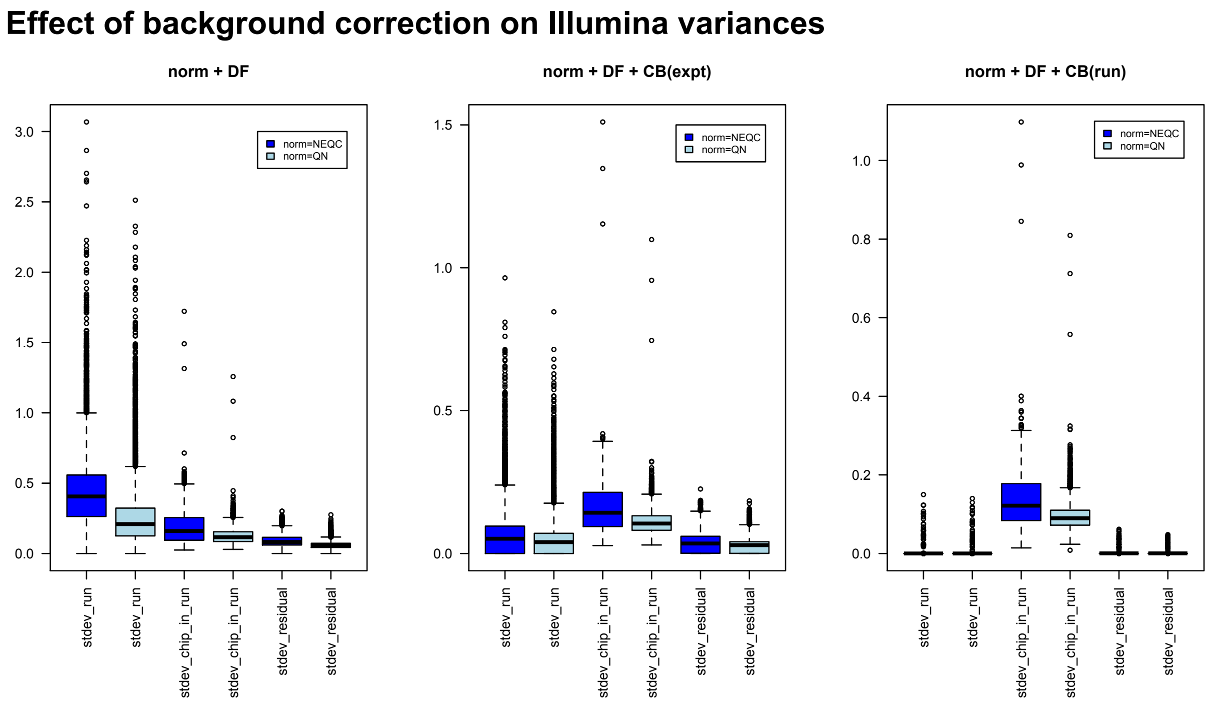
Effect of background correction on Illumina variances
norm + DF
norm + DF + CB(expt)
norm + DF + CB(run)
norm=NEQC
norm=QN
stdev_run
stdev_run
stdev_chip_in_run
stdev_chip_in_run
stdev_residual
stdev_residual
