# Supplementary figures and images for: Relative impact of key sources of systematic noise in Affymetrix and Illumina gene-expression microarray experiments
Source: BMC Genomics. 2011 Dec 1;12:589. doi: 10.1186/1471-2164-12-589 (PMC3269440; doi:10.1186/1471-2164-12-589)

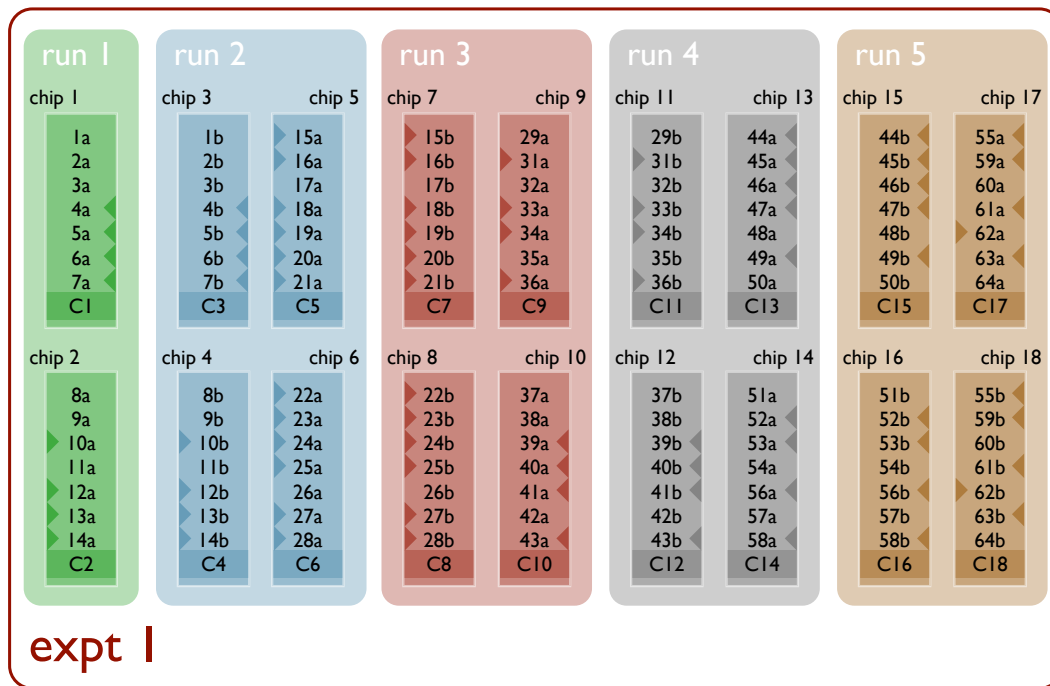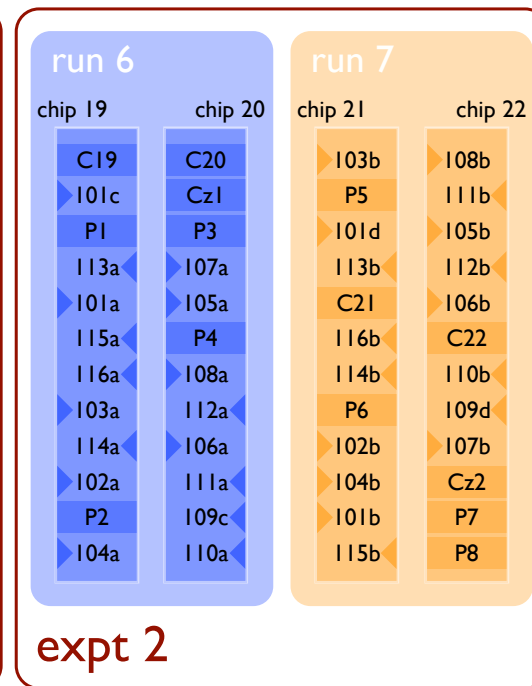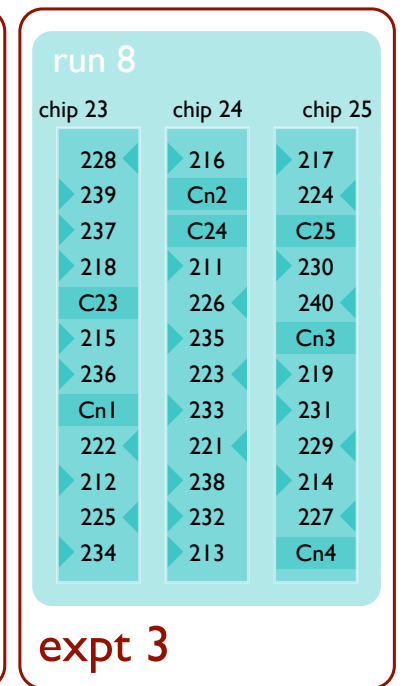

Supplement: Additional file 2 — Supplementary material S2. Illustration of our Illumina Ref-8 (experiment 1) and HT-12 (experiments 2 & 3) BeadChips, processed in eight batches (also referred to as 'runs') corresponding to the different days on which the samples were hybridised and scanned. UHRR samples are labelled as C1-25. Replicate breast tumour clinical samples are identified with a suffix of 'a' through 'd'. The pre- and post-treatment biopsy samples are identified by a triangle to the left and right of the sample IDs, respectively. [file 1471-2164-12-589-S2.PDF]

(Pearson) correlation with control sample fold-change

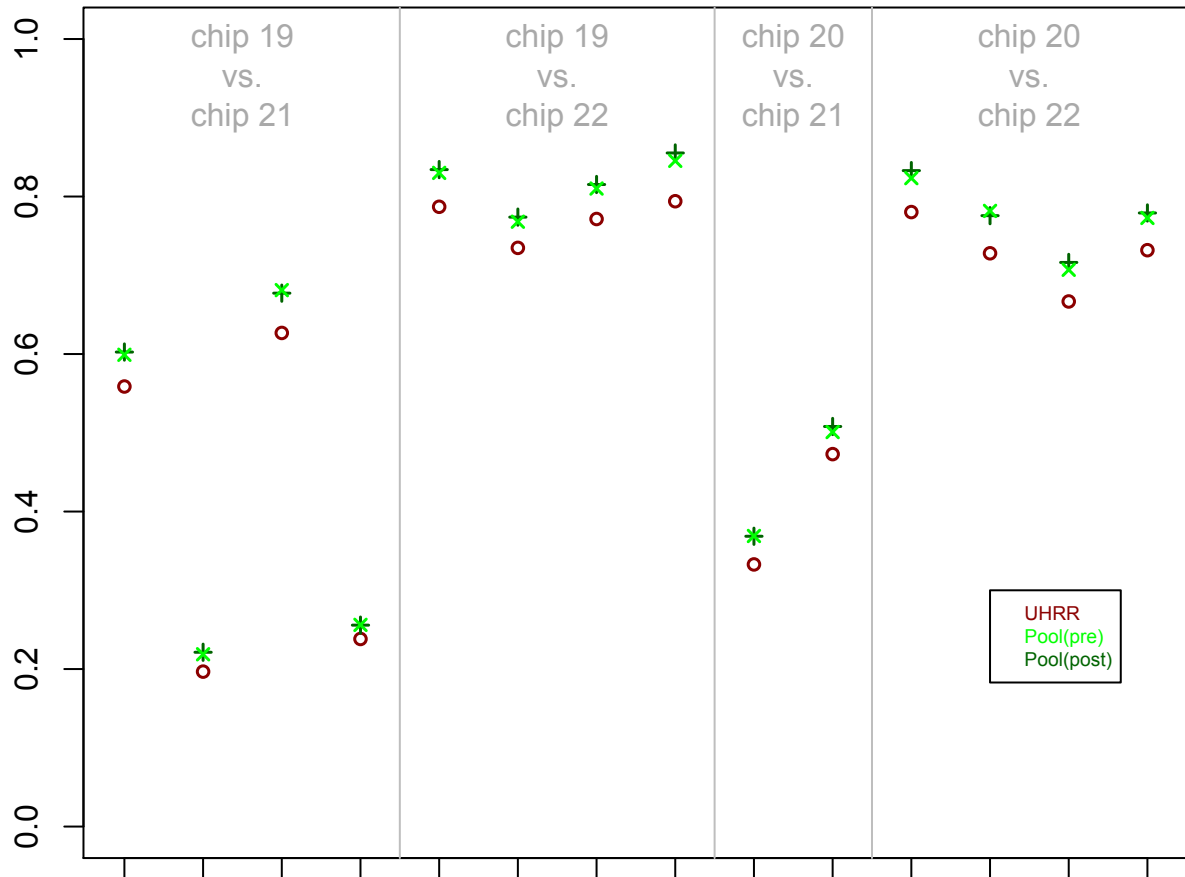

Supplement: Additional file 3 — Supplementary material S3. Correlation of expression change as a result of inter-run and inter-chip technical variation between UHRR and pooled controls with tumour duplicates. Tumour duplicates (individually plotted) are arranged on the x-axis to be close to others processed on the same BeadChip. UHRR and both types of pooled-control (comprised of pre- and post-treatment tumour RNA, respectively) are correlated more strongly with individual tumour duplicates in which one 'half' of the duplicate was processed on the outlying chip 22. Pooled-controls also consistently score slightly higher correlation than UHRR. [file 1471-2164-12-589-S3.PDF]

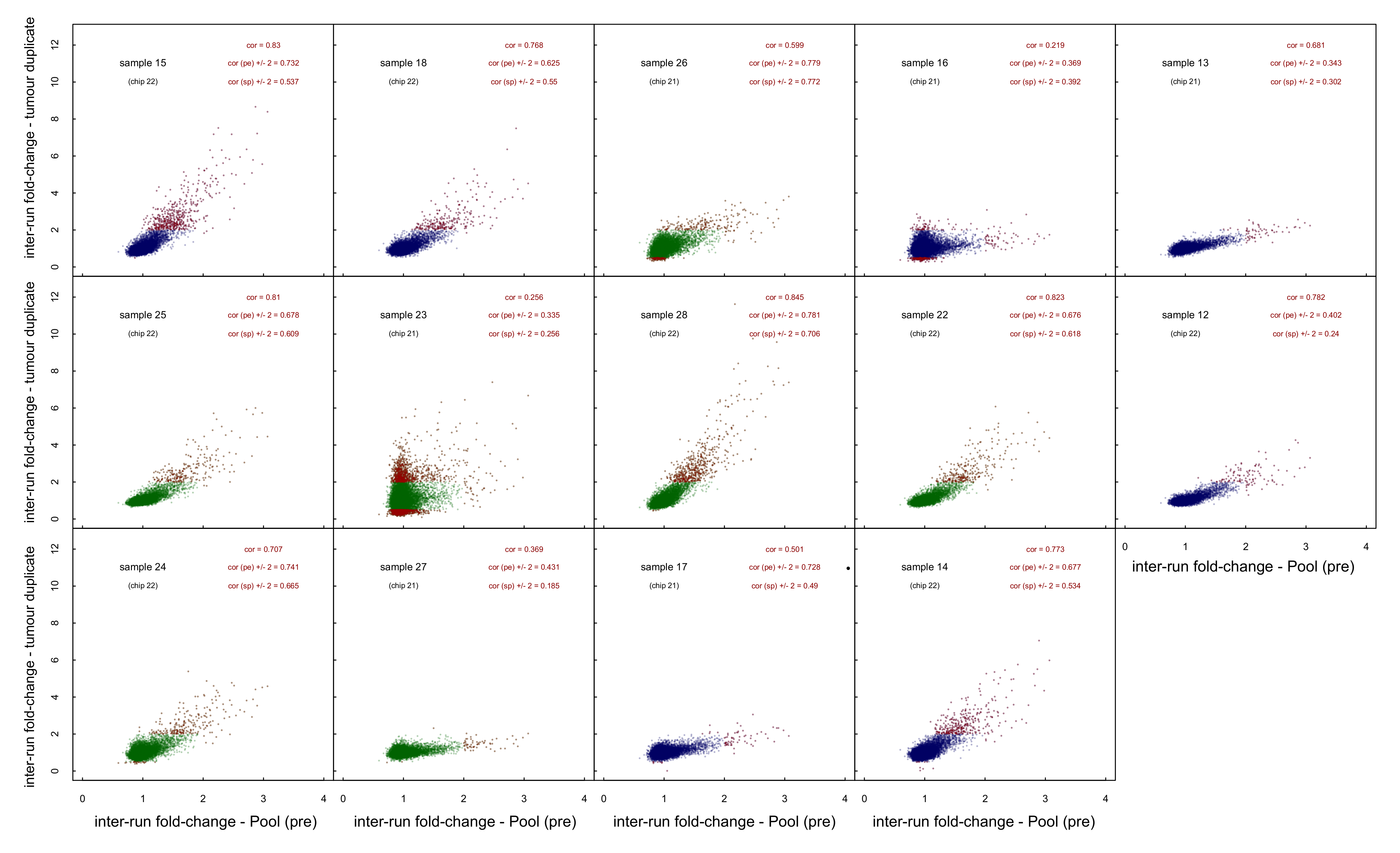

Supplement: Additional file 4 — Supplementary material S4. Scatter plots of fold-changes between tumour duplicates and replicate pools. These plots each show the magnitude of the change in expression between technical replicates introduced by the runs and show how well correlated such changes are between the Pool/tumour-duplicates. In the figure, the pre-treatment tumour duplicate are coloured blue and post-treatment are green; probes that are differentially regulated, up or down, at least two-fold due to the different runs are highlighted in each plot (red points). Note that most of the samples with a duplicate on chip 22 are subject to a greater magnitude of variation than samples with a duplicate on chip 21. [file 1471-2164-12-589-S4.PNG]

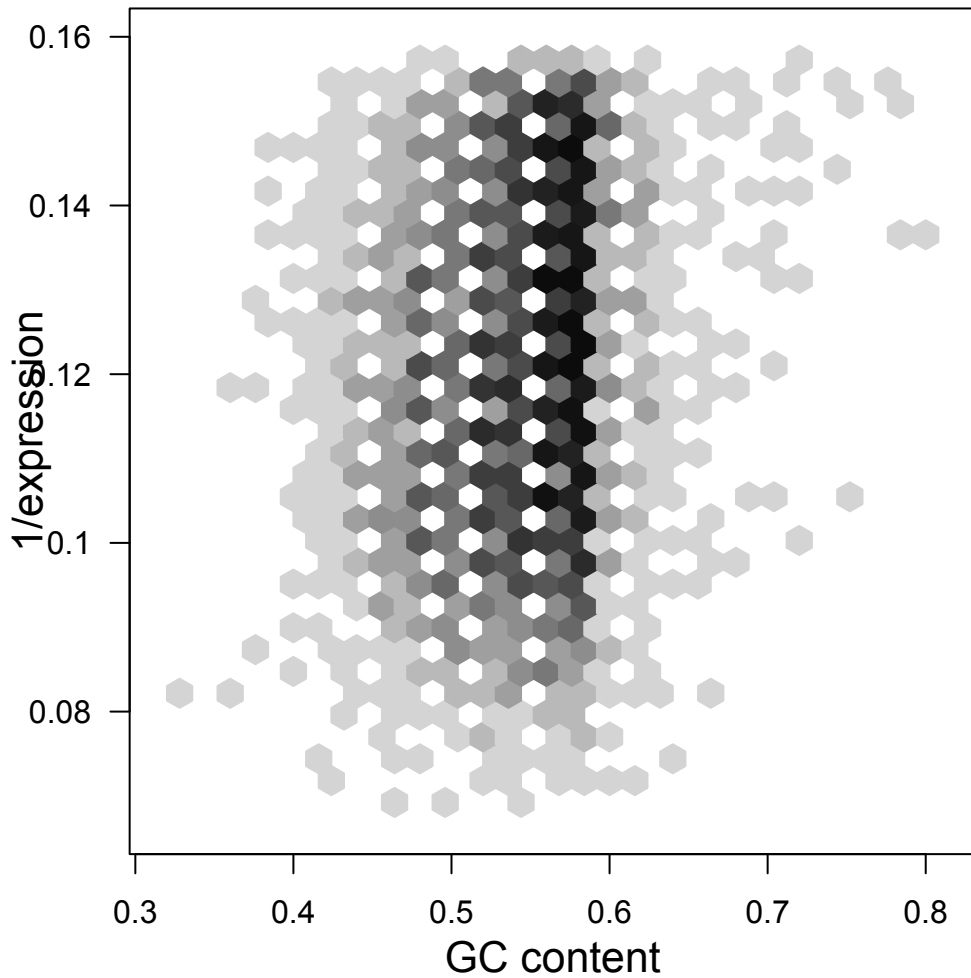

Counts

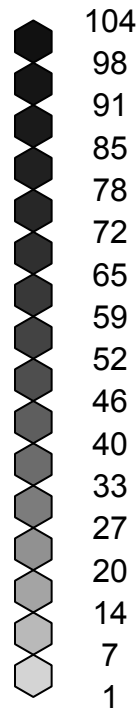

Supplement: Additional file 5 — Supplementary material S5. Plot of GC-content vs. (1/probe signal). [file 1471-2164-12-589-S5.PDF]

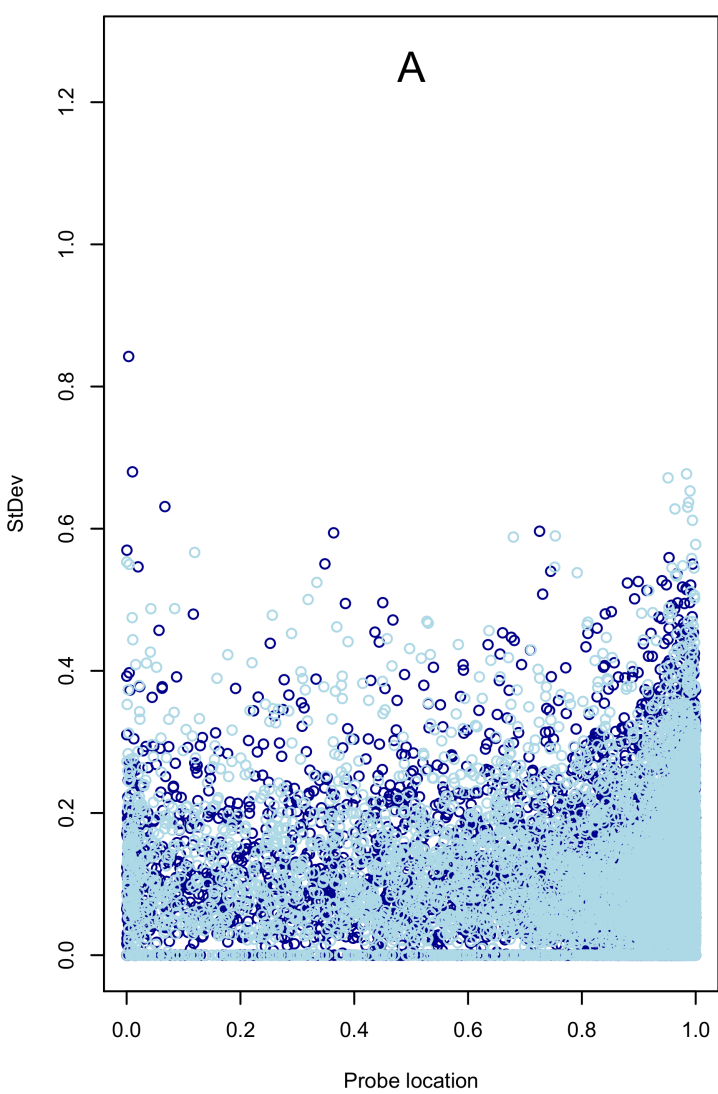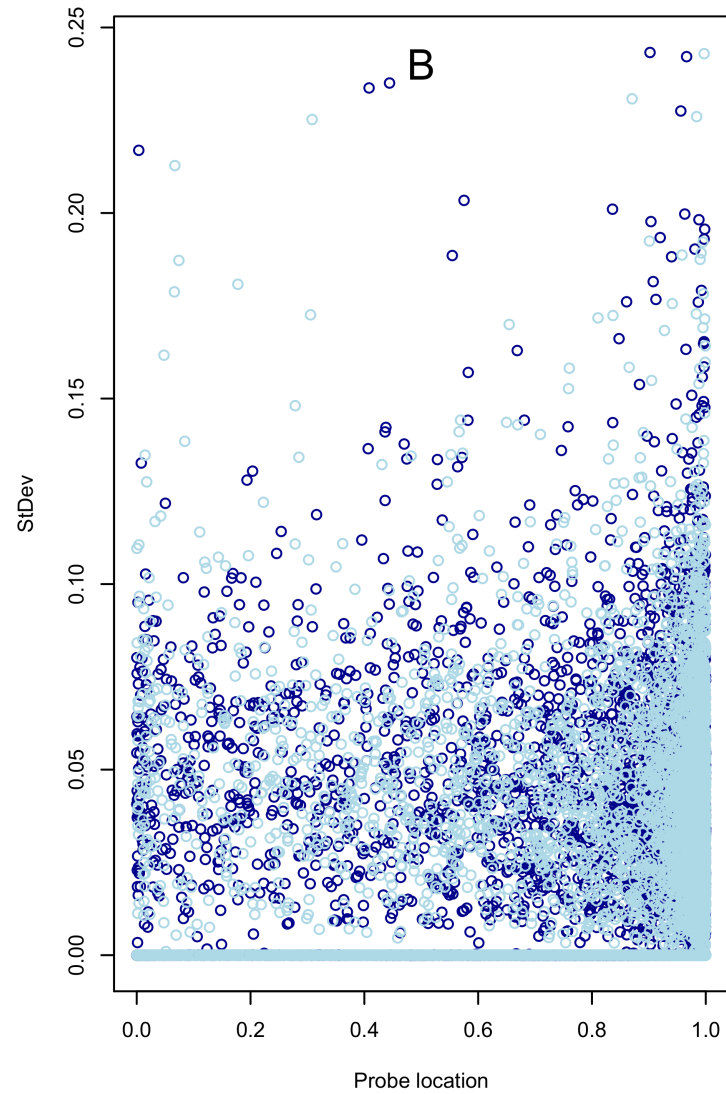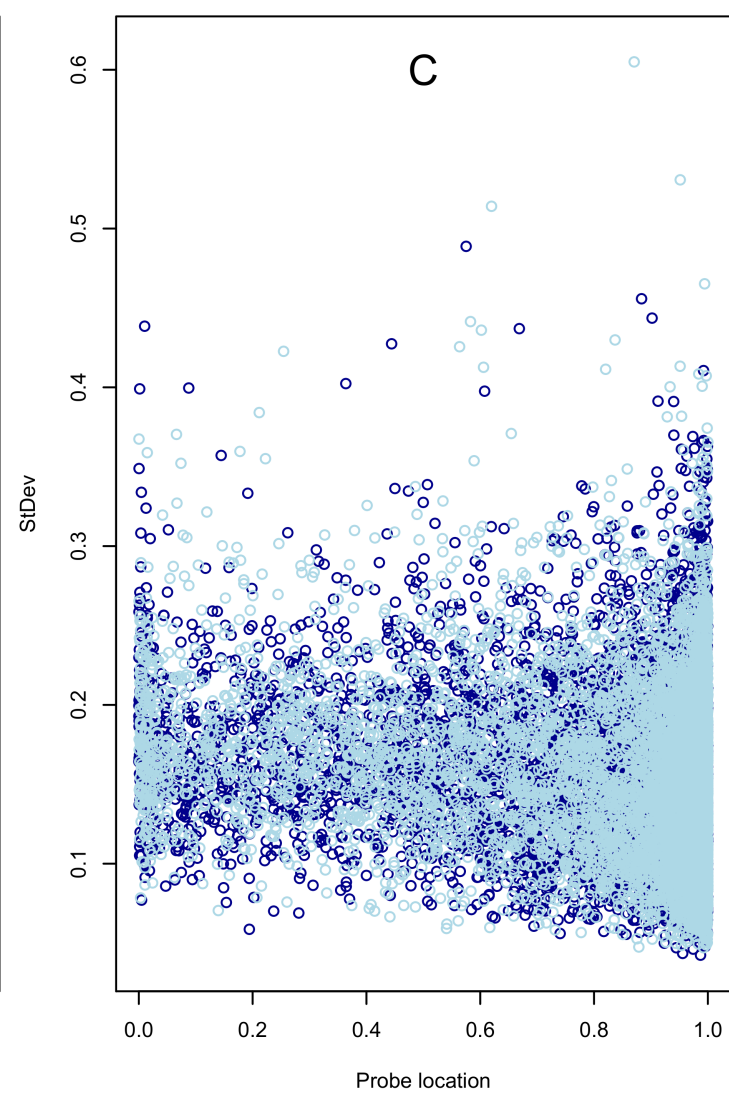

Supplement: Additional file 7 — Supplementary material S7. Probe position against probe standard deviation: Plots of probe position against probe standard deviation estimated at the inter-laboratory (A), inter-chip (B), and inter-array (C) levels in the MAQC Illumina dataset. Light and dark blue points again identify probes that mapped to the antisense and sense strands, respectively. [file 1471-2164-12-589-S7.PDF]
