# Supplementary material for: Relative impact of key sources of systematic noise in Affymetrix and Illumina gene-expression microarray experiments
Source: BMC Genomics. 2011 Dec 1;12:589. doi: 10.1186/1471-2164-12-589 (PMC3269440; doi:10.1186/1471-2164-12-589)
Supplement: Additional file 6 — Supplementary material S6. Distribution of MAQC Illumina probes as a fraction of target gene length. Light blue points are used for probes that mapped to the antisense DNA strand and dark blue for the sense strand. Left plot represents the fraction of target gene length in terms of 3' and 5' coordinates on each strand while the right plot is 'normalised' for the anti-sense strand and plots the fraction in terms of absolute position along the DNA molecule. [file 1471-2164-12-589-S6.PDF]

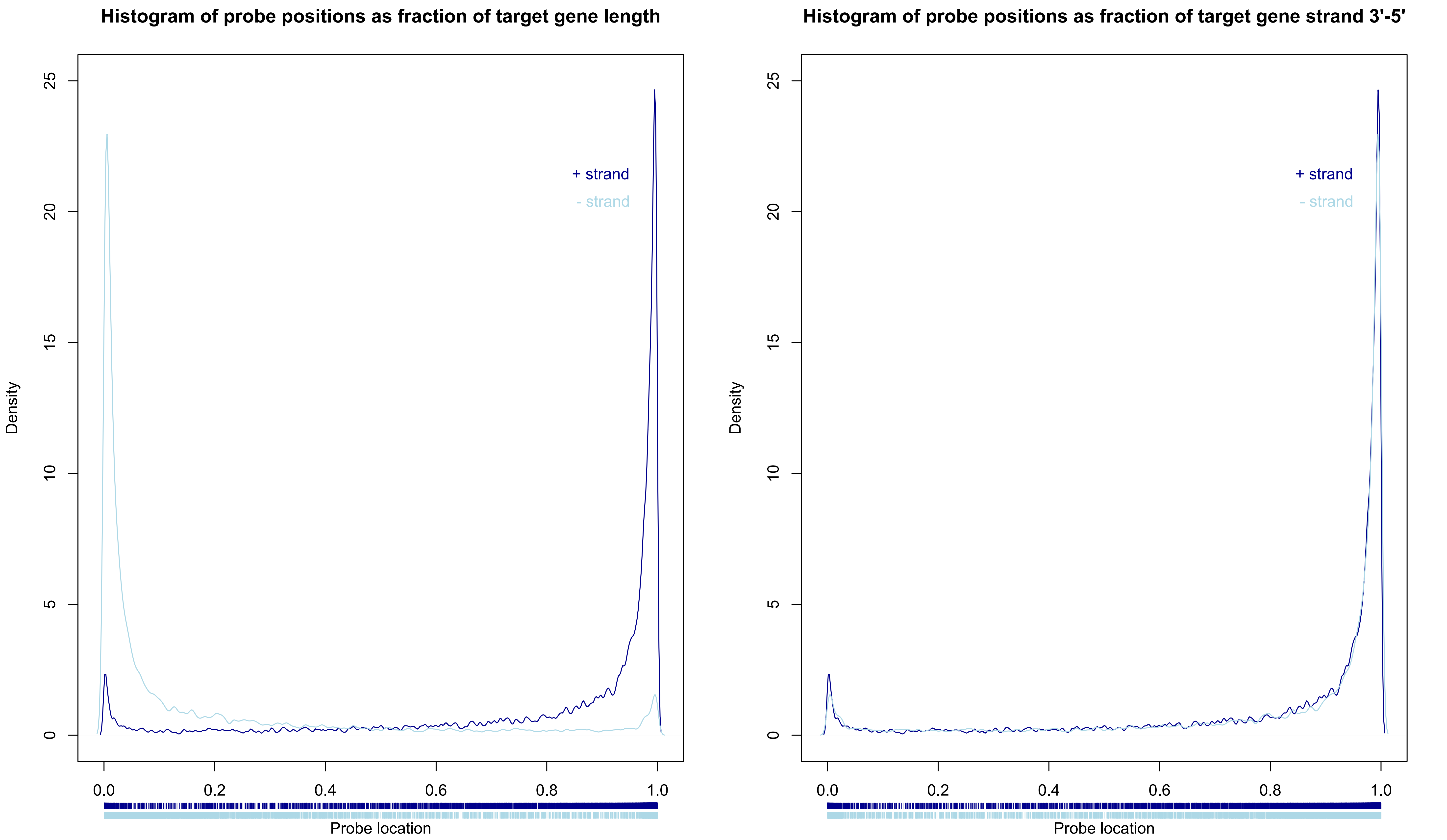
Histogram of probe positions as fraction of target gene length
Histogram of probe positions as fraction of target gene strand 3'-5'
+ strand
- strand
Density
Probe location
0
5
10
15
20
25
0.0
0.2
0.4
0.6
0.8
1.0
