# Supplementary material for: Relative impact of key sources of systematic noise in Affymetrix and Illumina gene-expression microarray experiments
Source: BMC Genomics. 2011 Dec 1;12:589. doi: 10.1186/1471-2164-12-589 (PMC3269440; doi:10.1186/1471-2164-12-589)

Signal distributions over all UHRR replicates

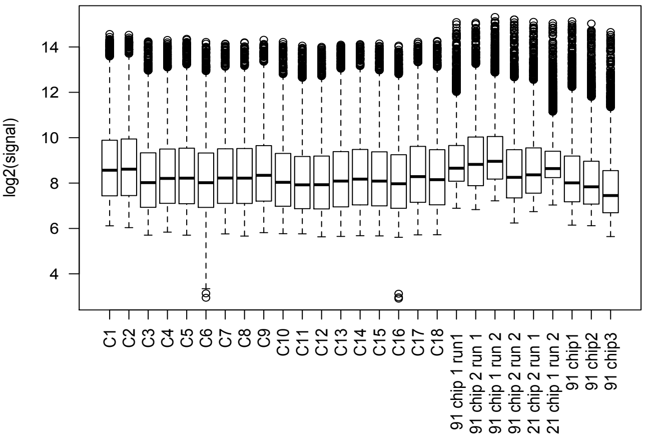

Signal distributions over all Affymetrix replicates

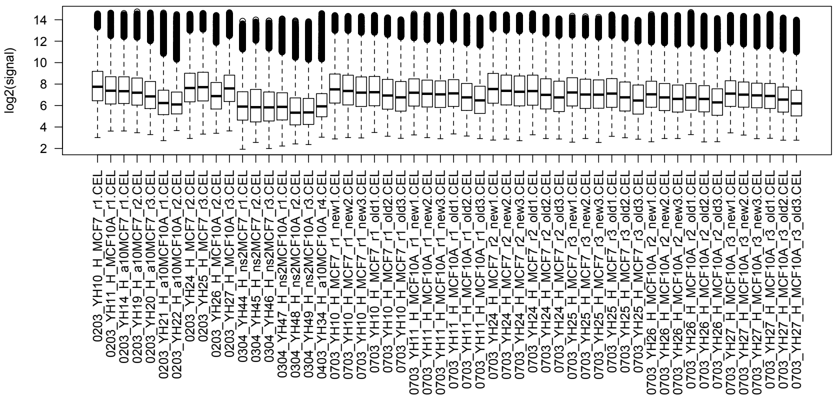

Ref8 - distribution of replicate bead count

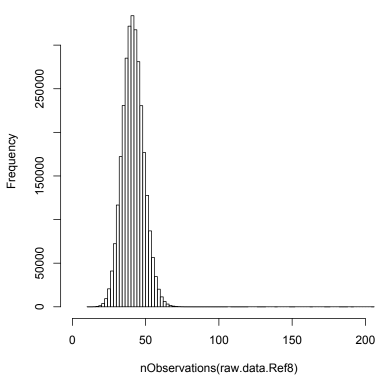

HT12 - distribution of replicate bead count

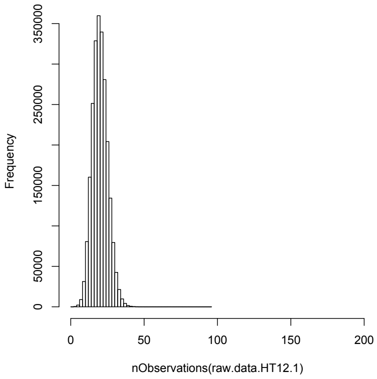

Ref8 - distribution of bead standard errors

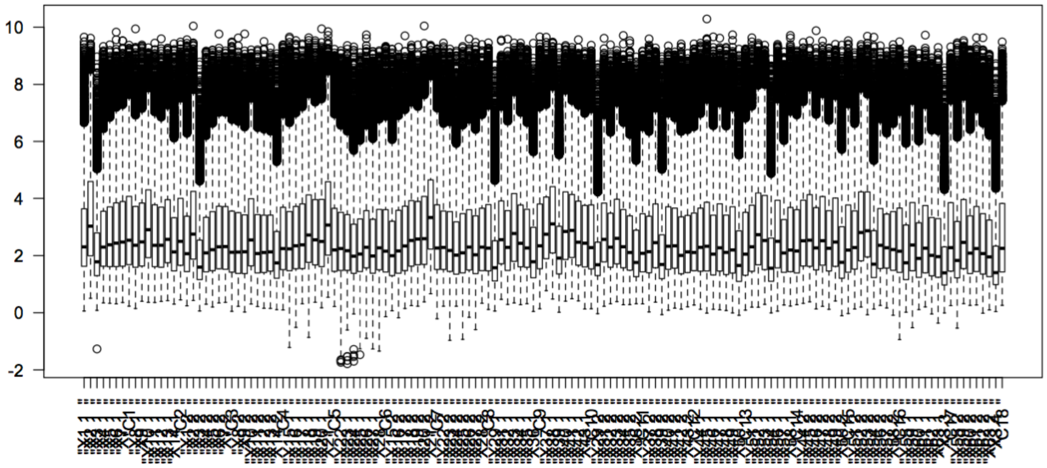

HT12 - distribution of bead standard errors

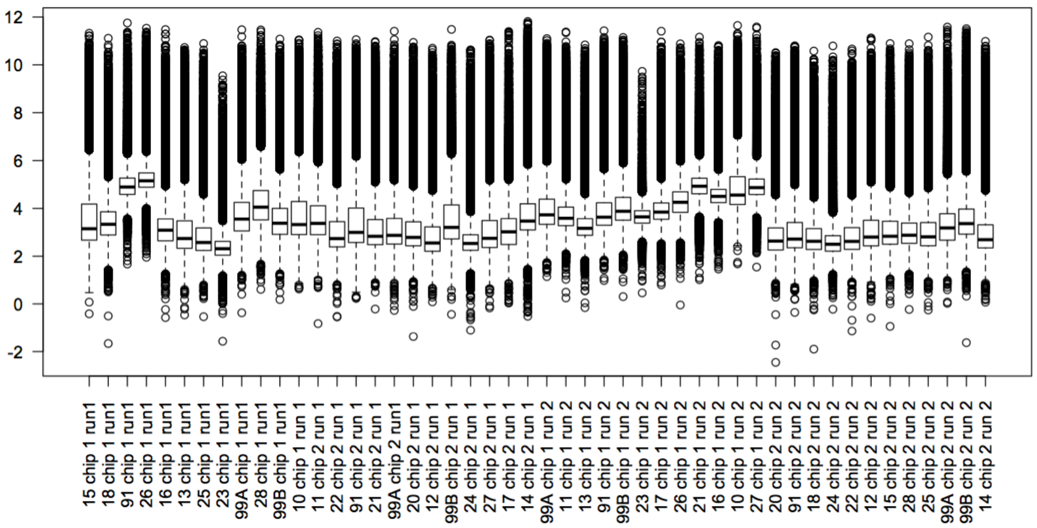

Supplement: Additional file 8 — Supplementary material S8. Quality control metrics over all of our Affymetrix and Illumina arrays including array-level intensity distributions, Illumina bead-standard errors, and bead-representation distributions between the two Illumina array-versions. [file 1471-2164-12-589-S8.PDF]
